# Atypical Neuroleptic Malignant Syndrome Without Hyperthermia: A Case Report

**DOI:** 10.7759/cureus.112233

**Published:** 2026-07-07

**Authors:** Abigael K Osena, Palak A Fichadia, Erick Melendez

**Affiliations:** 1 Psychiatry, Icahn School of Medicine at Mount Sinai, New York, USA

**Keywords:** autonomic instability, creatinine kinase, neuroleptic malignant syndrome (nms), recurrent psychosis, second generation antipsychotics

## Abstract

Neuroleptic malignant syndrome (NMS) is a rare but potentially life-threatening complication associated with antipsychotic use. Although classically characterized by hyperthermia, rigidity, altered mental status, and autonomic instability, atypical presentations lacking key features are increasingly recognized.

We present the case of a 21-year-old male admitted for psychosis and behavioral dysregulation who developed elevated creatine kinase (CK), hypertension, and tachycardia following treatment with multiple antipsychotics, including olanzapine and long-acting injectable aripiprazole. Notably, the patient remained afebrile and did not exhibit significant rigidity, complicating the diagnosis.

This case highlights the diagnostic challenges of atypical NMS and emphasizes the importance of maintaining clinical suspicion in patients with autonomic instability and elevated CK in the absence of classic features. Early recognition is essential to prevent morbidity and mortality.

## Introduction

Neuroleptic malignant syndrome (NMS) is a rare but potentially life-threatening neurologic emergency associated with dopamine receptor antagonists, including both first- and second-generation antipsychotics. The reported incidence ranges from 0.02% to 3% among patients receiving antipsychotic therapy [1,2]. 

NMS is classically defined by a tetrad of symptoms: altered mental status, muscle rigidity, hyperthermia, and autonomic instability. Heart, pulmonary, renal, musculoskeletal, and other systems are also affected by NMS complications. Usually, more than one-third of patients need to be treated in an intensive care unit [2]. In terms of management, all antipsychotics, dopamine-depleting, and dopamine-antagonistic medications should be stopped immediately, and intensive care should be given on a medical ward. All other psychotropic medications should be discontinued [2]. It is important to monitor and treat abnormalities in fluid/electrolyte balance, especially hyperkalemia, acid-base balance, cardiorespiratory function, rhabdomyolysis, renal function, level of awareness, and thromboembolic phenomena [2].

Rhabdomyolysis was the most common complication (30.1%). Sepsis (6.2%), acute renal injury (17.7%), acute respiratory failure (16.1%), and other systemic infections were additional frequent consequences. The unadjusted mortality rate was 5.6% [3]. However, atypical presentations lacking one or more of these hallmark features-particularly hyperthermia or rigidity-have been increasingly reported [4]. Milder or incomplete (“forme fruste”) presentations may occur, especially with second-generation antipsychotics or early recognition. In such cases, rigidity may be minimal or absent, and fever-traditionally considered a core diagnostic feature-may not be present. Isolated findings such as autonomic instability or elevated creatine kinase (CK) may not independently suggest NMS, contributing to diagnostic uncertainty [5].

We present a case of atypical NMS associated with antipsychotic polypharmacy, highlighting the challenges of diagnosis in the absence of classic clinical features.

## Case presentation

A 21-year-old male with a psychiatric history of bipolar I disorder (for the past four years), substance-induced psychotic disorder [during a few comprehensive psychiatric emergency program (CPEP) admissions], and alcohol use disorder (since he was 15 years old, four to five drinks/day) was admitted to an inpatient psychiatric unit for worsening mood symptoms for the past three weeks, psychosis, and inability to care for himself. He was initially on risperidone 1 mg twice daily, which was uptitrated to 3 mg daily during his previous admission, and he has been continued on the same for the past four years; he has no history of chronic medical conditions/trauma/substance use for other drugs, and has no known family history. Patient had never held a stable job and only worked odd jobs for the past few years, with his highest level of education being a completed gastroesophageal reflux disease (GED); he would socialize with his family and maintain a few close friendships. Spent most of his time in his room playing video games. According to the patient's family, he did not have any developmental delays. He had two prior psychiatric hospitalizations.

During a previous admission (approximately one year ago), he developed catatonia with markedly elevated CK (>22,000 U/L), suspected to represent atypical NMS, along with transaminitis. His mental status on current admission is shown in Table 1, as seen below.

On current admission, the patient presented with depressed mood, incongruent affect, passive suicidal ideation, and auditory hallucinations without delusions. His Bush-Francis Catatonia Rating Scale (BFCRS) score was 0, meaning he did not have catatonia at this point [6]. A complete physical examination did not show any pertinent positives, and a neurological examination presented with tremors, which were noted initially, raising concern for alcohol withdrawal; a chlordiazepoxide taper was initiated (starting with 100 mg STAT and a gradual reduction to 25mg in the span of five to six days) with minimal improvement in his tremors and he continued to experience auditory hallucinations, and these symptoms later improved with naltrexone 50mg once daily inthe next one week, which was tapered off and discontinued. Initial vitals showed mild tachycardia with HR ranging from 100 to 105, but this was controlled once he was started on chlordiazepoxide. Routine lab investigations, including complete blood count (CBC), complete metabolic profile (CMP), liver function tests (LFTs), and renal function tests (RFTs), were completed, and no pertinent positives were noted at that time; no imaging was conducted initially.

**Table 1:** Mental Status Upon Admission

Orientation	Alert
Appearance	Fairly groomed, wearing hospital clothes.
Attitude	Suspicious
Behavior	Bizarre
Eye Contact	Good
Speech	Normal in Rhythm, Volume, and Rate
Mood	“Miserable”
Affect	Incongruent: smiling while states to be sad and miserable.
Thought Process	Linear, goal directed.
Thought Content	No delusions elicited.
Suicidal Ideation	Patient endorsed: recent death wishes and suicide attempt but none at the moment, stated ''maybe in his forties''
Violent Ideation	Denied
Insight	Fair
Judgment	Poor
Impulse Control	Poor
Cognition	WNL

The patient was initially treated with risperidone, started on day one of admission, titrated to 3 mg daily for 10 days, but discontinued due to excessive sedation. He was transitioned to aripiprazole 10 mg daily, along with sertraline 25 mg once daily and naltrexone 50 mg once daily, with partial improvement in auditory hallucinations.

On hospital day 12, he received long-acting injectable naltrexone (380 mg) and aripiprazole (300 mg) with oral overlap of aripiprazole for two weeks; naltrexone was discontinued once he received the injection. The following day, he developed worsening command auditory hallucinations, which led to the patient showing more aggressive behavior towards other peers and staff members on the unit, and psychomotor slowing in terms of him not being able to complete his daily activities without assistance. Aripiprazole was increased to 15 mg in two divided doses of 10mg in the morning and 5 mg at night, and sertraline to 100 mg once daily in the morning. Due to agitation, olanzapine 5 mg sublingually every six hours was initiated.

Subsequently, in the next few days, the patient exhibited worsening disorganization, restlessness which lasted for most of the day, and confusion which was intermittent. His BFCRS score increased to 5, due to him developing autonomic instability, including hypertension (systolic blood pressure in the 150s mmHg) and tachycardia (heart rate 120-130 bpm). Laboratory evaluation revealed leukocytosis (12.07 × 10⁹/L) and elevated CK (1,207 U/L).

Given concern for evolving NMS, all of the oral psychotropic medications were discontinued on hospital day 22, and lorazepam 1 mg twice daily was initiated for suspected catatonia. The patient initially received 1 L of intravenous fluids (IVF) [normal saline solution (NSS)], then an additional 2L of IVF (NSS) on a different day. Despite these interventions, the patient required multiple intramuscular medications that included: lorazepam 2 mg IM STAT - received three doses (day 17 of hospitalization), lorazepam 1 mg IM STAT- received only one dose (day 18 of hospitalization), Benadryl 50 mg IM STAT - received three doses (day 18 of hospitalization), olanzapine 5 mg IM STAT - received four doses (day 17 of hospitalization), Benadryl 25 mg IM STAT - received three doses (day 18 of hospitalization), haloperidol 5 mg IM STAT - received two doses (day 18 of hospitalization), chlorpromazine 100 mg IM STAT - received one dose (day 21 of hospitalization), Benadryl 50 mg IM STAT - received one dose (day 21 of hospitalization), haloperidol 5 mg IM STAT - received three doses (day 21 of hospitalization), lorazepam 2 mg IM STAT - received three doses (day 21 of hospitalization)and oral medications as appropriate (lorazepam 2 mg tab q8h for acute anxiety, Benadryl 50 mg tab q12h for extra-pyramidal symptoms (EPS)/anxiety, olanzapine 5 mg tab q8h for acute agitation, and haloperidol 5 mg tab q8h for acute agitation) and physical restraints due to worsening agitation. He remained afebrile but continued to demonstrate persistent hypertension [systolic blood pressure (SBP) 160s mmHg] and tachycardia (HR 110s bpm). CK increased to 4,079 U/L. Additional laboratory findings included C-reactive protein 61.8 mg/L and aspartate aminotransferase (AST) 80 U/L, noted on day 16 of hospitalization. Olanzapine was discontinued due to concern for NMS. Tables 2, 3, 4 show a summary of all the abnormal labs during this current admission.

**Table 2:** Serial Complete Blood Count During Hospitalization RBC: red blood cell count, WBC: white blood cell count.

Parameter	Reference Range	Day 1	Day 16	Day 17	Day 19	Day 22	Day 29
WBC (×10³/µL)	4.80–10.80	9.87	13.07↑	7.64	6.57	7.19	5.75
RBC (×10⁶/µL)	4.70–6.10	4.81	4.93	4.91	4.46↓	4.23↓	4.57↓
Hemoglobin (g/dL)	14.0–18.0	15	15.3	15.3	13.8↓	13.1↓	14
Hematocrit (%)	42.0–52.0	44	47.7	44.7	43.8	39.7↓	42.7
Platelets (×10³/µL)	150–450	285	291	370	290	257	420
Neutrophils (%)	44–70	71.3↑	66.7	64.3	63.1	63.8	58.1
Lymphocytes (%)	20–45	15.2↓	15.7↓	20	22.8	22.3	27.3
Monocytes (%)	2–10	12.8↑	15.5↑	12.3↑	11.0↑	8.8	10.1↑
Eosinophils (%)	1–4	0.1↓	1.4	2.4	2	4.2↑	3.7
Basophils (%)	0.2–1.8	0.4	0.5	0.7	0.9	0.6	0.5

**Table 3:** Serial Chemistry Panel and Liver Function Tests During Hospitalization CO_2_: carbon dioxide; AST: aspartate aminotransferase; ALT: alanine aminotransferase.

Parameter	Reference Range	Day 1	Day 2	Day 14	Day 16	Day 17	Day 19	Day 21	Day 22	Day 29	Day 34
Sodium (mmol/L)	136–145	139	137	138	136	138	139	141	142	138	139
Potassium (mmol/L)	3.5–5.1	4.4	4.8	4.6	4.6	4.6	3.9	4.7	3.6	4.3	3.8
Chloride (mmol/L)	98–108	100	100	101	98	101	104	109↑	107	101	101
CO₂ (mmol/L)	22–29	19↓	24	22	23	22	20↓	19↓	21↓	25	24
BUN (mg/dL)	6–23	14	12	11	10	13	9	12	10	8	8
Creatinine (mg/dL)	0.70–1.20	1.37↑	1.12	1.19	1.15	1.14	0.94	1.12	0.91	1.11	1.1
Glucose (mg/dL)	74–106	108↑	107↑	113↑	104	96	103	90	127↑	90	90
Calcium (mg/dL)	8.6–10.3	10.2	9.8	10.1	9.7	9.5	9.2	8.6	9	10	9.4
Albumin (g/dL)	3.5–5.2	4.8	4.6	4.8	4.3	4.2	4.1	3.7	3.4↓	3.9	3.9
Total Protein (g/dL)	6.6–8.7	8.5	7.5	8.5	7.6	7.9	7	6.7	6.6	7.2	7.4
AST (U/L)	5–40	54↑	52↑	21	38	39	32	66↑	80↑	24	21
ALT (U/L)	0–41	29	31	27	22	28	22	24	30	37	28
Alkaline phosphatase (U/L)	40–129	89	89	107	90	91	84	79	87	84	104
Total bilirubin (mg/dL)	0–1.2	0.9	0.6	0.2	0.5	0.4	0.5	0.3	0.3	0.2	<0.2

**Table 4:** Serial Neuroleptic Malignant Syndrome–Related Laboratory Investigations TSH: thyroid stimulating hormone; HDL: high-density lipid; LDL: low-density lipid.

Parameter	Reference Range	Day 1	Day 16	Day 17	Day 19	Day 21	Day 22	Day 29	Day 34
Creatine kinase (U/L)	20–190	—	1,207↑	843↑	950↑	3,113↑	4,079↑	254↑	166
High-sensitivity Troponin T (ng/L)	0–22	—	<6	—	<6	<6	—	6	—
High-sensitivity CRP (mg/L)	≤5	—	—	—	—	65.6↑	61.9↑	—	—
Magnesium (mg/dL)	1.6–2.6	—	—	—	2	1.9	—	—	—
Phosphorus (mg/dL)	2.5–4.5	—	—	—	3.4	3.5	—	—	—
TSH (µIU/mL)	0.27–4.20	—	1.24	—	—	—	—	—	—
Hemoglobin A1c (%)	4.0–5.6	5.5	—	—	—	—	—	—	—
Cholesterol (mg/dL)	≤240	—	168	—	—	—	—	—	—
HDL (mg/dL)	35–65	—	49	—	—	—	—	—	—
LDL (mg/dL)	60–129	—	108	—	—	—	—	—	—
Triglycerides (mg/dL)	≤150	—	53	—	—	—	—	—	—

Haloperidol was briefly reintroduced due to availability in both oral and intramuscular formulations. The patient was on haloperidol 5 mg tab every eight hours as needed for agitation from day one to 15 of hospitalization. It was shifted to Zyprexa 5mg tab SL q6 for agitation due to poor response to haloperidol; it was used as needed medication for psychosis/agitation - last given on day 15, reintroduced on day 20 of hospitalization. Patient was on haloperidol 2mg tablet twice a day, due to its availability in oral and intramuscular form and to minimize polypharmacy and the risk of NMS, discontinued on day 22 of hospitalization due to poor response, as he remained disorganized and illogical.

Benzodiazepine therapy was adjusted but later tapered due to concern for worsening delirium. The patient’s clinical picture was complicated by ongoing agitation and disorganization, likely exacerbated by polypharmacy. Patient was started on lorazepam 1 mg tablet twice a day on day 16 increased to 1 mg tablet thrice a day on day 17, discontinued standing lorazepam on day 20 due to poor response - remained agitated, disorganized, started on clonazepam 1 mg tablet every 12 hours on day 21 (longer acting), discontinued clonazepam 1 mg tablet every 12 hours on day 24 to avoid worsening confusion and rebound agitation.

Chlorpromazine was initiated with gradual improvement in behavioral control, patient was started on chlorpromazine 50 mg tab every 12 hours for his psychosis and chlorpromazine 50 mg tab every eight hour for agitation on day 22 (decreased on day 31, from chlorpromazine 50 mg tab thrice a day to 50 mg tab twice a day, decreased on day 34 to chlorpromazine 50 mg tab nightly, discontinued on day 36 of hospitalization), since patient showed further poor response with the haloperidol and patient was noted with improved behavioral control on Thorazine, it was increased to 50 mg tablet to thrice a day on day 23.As per staff, less agitation and disorganized behavior. Did not require further STAT medications after receiving chlorpromazine. 

Delirium tremens was considered given the history of alcohol use and presence of tremors; however, normal blood alcohol levels, predominant psychotic features, and clinical improvement with antipsychotics made this diagnosis less likely. 

Given prior stability on aripiprazole, chlorpromazine was tapered, and aripiprazole was reintroduced and titrated to 15 mg daily; aripiprazole 10 mg daily was started on day 28 of hospitalization and increased to 15 mg daily on day 31 of hospitalization. The patient demonstrated progressive improvement in orientation and psychotic symptoms. On day 34 of hospitalization, his level of orientation improved (oriented to self, place, date) gradually, and he was noted to socialize with peers and engage in group activities. He also reported a gradual decrease in disturbance from voices. Patient denied suicide or homicide ideation; no episode of agitation or self-injurious behavior in the unit. Initial BFCRS was 5 - mild stupor (+1), mild mutism (+1), mild staring (+1), moderate withdrawal (+2). BFRCS was 0 before discharge.

CK levels normalized to 166 U/L before discharge. On improvement, the patient's mental status before discharge is seen in Table 5, as seen below, total of 38 days of hospitalization; patient was discharged w/ aripiprazole 15mg daily and was scheduled for a follow-up after three days at the outpatient clinic, with onTrackNY (OTNY) services.

**Table 5:** Mental Status Examination upon Discharge

Orientation	Alert
Appearance	Casually groomed
Attitude	Cooperative
Behavior	Within normal limits
Eye Contact	Good
Speech	Normal rate, rhythm and volume
Mood	Euthymic
Affect	Appropriate
Thought Process	Linear
Thought Content	None
Suicidal Ideation	Patient denied
Violent Ideation	Patient denied
Perceptual Disturbances	Patient denied
Insight	Good
Judgment	Good
Impulse Control	Good
Cognition	UTA

His level of orientation improved gradually, and he was noted to be leaving his room more often to socialize with peers and engage in group activities. Patient responded well to this treatment - medications, milieu, and structured setting; became much more organized, no longer psychotic or depressed. No episode of agitation or self-injurious behavior in the unit for 13 days.

## Discussion

NMS associated with second-generation antipsychotics may present atypically, often lacking hyperthermia or rigidity [5]. A retrospective analysis of 26 case reports found that atypical NMS was commonly associated with clozapine, olanzapine, aripiprazole, and quetiapine. Notably, clozapine and quetiapine were frequently associated with absent rigidity, while aripiprazole and clozapine were often associated with the absence of hyperthermia [5].

Although diagnostic criteria vary, there is general agreement that core features include altered mental status, autonomic instability, rigidity, and hyperthermia [7]. In this case, the patient exhibited autonomic instability, altered mental status, and elevated CK but lacked hyperthermia and significant rigidity, consistent with an atypical presentation.

While dopamine receptor blockade remains the primary proposed mechanism, atypical presentations suggest a more complex pathophysiology involving serotonergic, adrenergic, and cholinergic systems [8]. 

Atypical presentations indicate that NMS pathophysiology extends beyond simple dopamine D2 receptor blockade in the nigrostriatal and hypothalamic pathways. Evidence implicates interplay with the serotonergic system (notably 5‑HT2A modulation affecting thermoregulation and muscle tone), adrenergic signaling (altered α/β activity influencing autonomic instability, blood pressure lability, and hyperthermia), and cholinergic tone (anticholinergic burden compounding hyperthermia and delirium) [8].

This network-level dysregulation helps explain cases triggered by atypical antipsychotics, mixed psychopharmacology, or abrupt dopaminergic withdrawal, where pure dopaminergic antagonism seems insufficient to account for the full clinical spectrum. Concurrently, dysregulation of the sympathoadrenal axis likely amplifies metabolic heat production, rigidity-associated ATP consumption, and rhabdomyolysis via catecholamine surges, creating a self-reinforcing loop of hyperthermia and autonomic dysfunction [8].

Genetic predisposition-potentially involving variants in dopamine receptors, ryanodine receptor (RYR1)-related calcium handling, or malignant hyperthermia susceptibility pathways-may lower the threshold for triggering events, aligning with observed interindividual vulnerability. Together, these mechanisms support a multi-system model in which dopaminergic blockade initiates risk, while serotonergic, adrenergic, cholinergic, sympathoadrenal, and genetic factors determine severity, phenotype, and treatment responsiveness [8].

Dysregulation of the sympathoadrenal system and genetic predisposition may also contribute [9]. CK is elevated in NMS because of severe generalized skeletal muscle rigidity and sustained muscle contraction resulting from central dopamine (D2) receptor blockade in the hypothalamus and basal ganglia. [9]

The intense muscle rigidity increases metabolic activity, leading to ATP depletion, muscle ischemia, and muscle fiber necrosis (rhabdomyolysis). As skeletal muscle cells are damaged, intracellular enzymes and proteins, including CK, myoglobin, lactate dehydrogenase (LDH), potassium, and phosphate, leak into the bloodstream. CK levels typically exceed 1,000 IU/L and may reach 10,000-100,000 IU/L in severe cases [9].

Elevated CK is an important laboratory marker of muscle injury and correlates with disease severity and the risk of complications such as rhabdomyolysis and acute kidney injury. However, it is not specific for NMS and may also be elevated in other conditions associated with muscle injury [9].

The most likely etiology in this case was antipsychotic polypharmacy, including concurrent use of long-acting injectable aripiprazole and olanzapine, along with rapid medication adjustments. This is consistent with prior literature suggesting increased risk of NMS when multiple antipsychotics are used concurrently [4]. 

The development of NMS is associated with several drug-related, patient-related, and environmental risk factors. Drug-related factors include the use of high-potency first-generation antipsychotics such as haloperidol and fluphenazine, rapid dose escalation, high antipsychotic doses, depot (long-acting injectable) formulations, concurrent use of multiple dopamine-blocking medications, and abrupt withdrawal of dopaminergic drugs such as levodopa or bromocriptine in patients with Parkinson disease [4].

Patient-related risk factors include a previous history of NMS, young adult age, male sex, agitation, catatonia, dehydration, malnutrition, iron deficiency, physical restraint, intellectual disability, Parkinson disease, organic brain disorders, and mood disorders. Medical conditions such as infections, electrolyte disturbances, hyperthyroidism, trauma, surgery, heat exposure, and substance use, particularly cocaine, may further increase the risk. Genetic susceptibility has also been suggested, although the exact mechanisms remain under investigation [4].

Table 6 shows the various differential diagnoses and their associated signs and symptoms. The following table outlines the principal differential diagnoses of NMS and their associated signs and symptoms. Given the significant clinical overlap with conditions such as malignant catatonia, malignant hyperthermia, sepsis, central nervous system (CNS) infection, heat stroke, withdrawal syndromes, and parkinsonism, accurate differentiation is essential to ensure timely diagnosis and appropriate management.

**Table 6:** Differential Diagnoses CNS: central nervous system; EtOH: ethanol; Benzo: benzodiazepines.

Condition	Trigger	Onset	Muscle Findings	Key Distinction
Neuroleptic Malignant Syndrome	Dopamine antagonists	Days–weeks	Lead-pipe rigidity	↑ CK, no clonus
Serotonin Syndrome	Serotonergic drugs	Hours	Clonus, hyperreflexia	Rapid onset
Malignant Hyperthermia	Anesthesia	Minutes–hours	Rigidity	Operating room / post-op
Malignant Catatonia	Psychiatric illness	Days	Rigidity	No neuroleptic use
Sepsis	Infection	Variable	None	Infectious source
CNS Infection	Infection	Hours–days	Neck stiffness	Abnormal Cerebrospinal fluid
Heat Stroke	Heat/exertion	Acute	None	Hot, dry skin
Withdrawal (EtOH / Benzo)	Medication cessation	Hours–days	Tremor	Withdrawal history
Parkinsonism–Hyperpyrexia	Levodopa withdrawal	Days	Rigidity	Parkinson’s disease

## Conclusions

NMS is associated with significant morbidity and mortality, and atypical presentations may delay diagnosis. This case highlights the importance of maintaining a high index of suspicion for NMS in patients with autonomic instability and elevated CK, even in the absence of hyperthermia or rigidity. Early recognition and prompt intervention are critical to improving clinical outcomes, particularly in the setting of antipsychotic polypharmacy.
